# A Psychological Point of View on Endometriosis and Quality of Life: A Narrative Review

**DOI:** 10.3390/jpm14050466

**Published:** 2024-04-28

**Authors:** Elisa Farenga, Matteo Bulfon, Cristiana Dalla Zonca, Costanza Tersar, Giuseppe Ricci, Giovanni Di Lorenzo, Andrea Clarici

**Affiliations:** 1 S.C.U. Obstetrics and Gynaecology Clinic, Institute for Maternal and Child Health, IRCCS “Burlo Garofolo”, Via dell’Istria, 65, 34137 Trieste, Italy; 2 Department of Medical, Surgical and Health Sciences, University of Trieste (UniTS), Cattinara Hospital, Strada di Fiume, 447, 34149 Trieste, Italy; clarici@units.it; 3 Centro Formazione e Ricerca in Psicoterapia a Orientamento Psicoanalitico, Via Antonio Canova, 2, 34129 Trieste, Italy

**Keywords:** psychological factors, endometriosis, quality of life, pain

## Abstract

Endometriosis is a chronic gynecological disorder with a multifactorial etiology that has not yet been fully elucidated. What is known, however, are the pathological tissue dynamics that lead to the complex symptoms that women suffer from. The known symptoms are mainly fertility problems and pain. Both dimensions have an impact that varies from case to case, but that is certainly decisive concerning a woman’s health, specifically by affecting the overall quality of life (QoL). In this publication, we will deal with the descriptive aspects of endometriosis’s pathology and then present a review of the aspects impacting QoL and their psycho-social consequences. Finally, the experience of pain in the context of the mind–brain–body relationship will be discussed, describing the complexity of this dimension and emphasizing the importance of a multi-professional approach that considers the relevance of the contribution that a psychotherapy intervention based on up-to-date neurobiological models can make for women with endometriosis. A review of the literature and current knowledge on the neural and psychological aspects of pain lead to the conclusion that it is of the utmost importance to provide informed psychological support, alongside medical treatments and sexual counseling, to patients with endometriosis.

## 1. Introduction

Endometriosis is defined as a gynecological disease of uncertain etiology. It is a chronic and recurrent inflammatory disease, characterized by the presence of endometrial tissue in ectopic sites. The most typical symptoms are pain and infertility [1].

In pelvic localizations, lesions are more frequently found in the ovaries, with adhesion between organs creating a pro-inflammatory pattern and organ obstruction, with a risk of compromising the woman’s fertility [2] and pain. On the other hand, extragenital localizations are often associated with intestinal and urinary tract localizations, leading to organ damage and pain symptoms or affecting the urinary tract.

According to Ministry of Health estimates, 3 million people in Italy are affected by endometriosis, 10–15% of whom are women of childbearing ages [3].

At the time of diagnosis, the highest incidence of the disease is between the ages of 25 and 45, although it can also appear in younger ages [1,2,3]. Specifically, endometriosis affects 35–50% of women who report chronic pelvic pain, infertility, or both [3]. In women with dysmenorrhea and pelvic pain, the incidence varies between 40% and 60%, while in women with infertility, it varies between 20% and 30%. The severity of symptoms and the possibility of obtaining a diagnosis increase with age [4].

Despite these generally accepted data, the true prevalence of endometriosis in women of a childbearing age remains difficult to establish, partly due to the difficulty in arriving at a diagnosis. However, it is worth noting that recent local research showed that the incidence of endometriosis is 111 cases per 100,000 women between 15 and 50 years [5,6]. The wide incidence of endometriosis requires prompts to analyze its impact on the quality of life of women and their social environment.

The articles for this review were chosen by drawing from two databases: MDPI Open Access Journals and NIH—National Library of Medicine. The studies were selected taking into account the national (Italy) and regional (Friuli Venezia Giulia region) context, with the intent to represent the current state of the pathology and research on it. The articles were then selected with an aim to investigate the quality of life of women with endometriosis, especially in relation to psychological factors, by choosing the most recent sources that dealt with endometriosis in its etiological and symptomatological aspects.

After a broad introduction on the main aspects that characterize endometriosis, this study intends to deal with the psychological aspects that impact on QoL, in particular the experience of pain, encompassing its various psycho-somatic aspects.

## 2. Endometriosis: The Troubles in Reaching a Diagnosis

Although there are several theories explaining endometriosis [7,8], the causes and pathogenetic mechanisms are still under debate. It is believed that the onset of the disease and its evolution are variable; it could be the consequence of a complex series of events involving a genetic predisposition, anatomical factors, abnormalities of the immune system, and environmental influences.

All these elements act in concert as concauses; the disease could have a persistent course over time in its severe or mild forms or, in other cases, disappear altogether.

One of the variables that make the correct diagnosis of endometriosis difficult is its clinical variability: today, the time between the first appearance of symptoms and diagnosis varies between 8 and 10 years [1]. This is particularly so because symptoms are non-specific, and manifestations are heterogeneous [1]. This is determined on the one hand by the different localization of endometrial tissue and on the other by the severity or extent of the disease itself. It is estimated that 25% of cases are asymptomatic, and it is sometimes the case that the disease is only diagnosed during examinations for infertility-related problems [4].

The genetic component seems to influence the onset of the disease in 50% of cases, while the remaining 50% depend on environmental influences [9]. The two components, genetic and environmental, could trigger the disease by interacting with each other or by acting as independent factors.

Research on environmental factors shows that various factors can influence DNA methylation, and prenatal exposure to stress or other toxic environmental elements can contribute to the pathogenesis of endometriosis [7]. Indeed, toxic substances at the cellular level have also been found in adult patients: in-depth analysis of the cellular physiology of endometrial tissue and macrophages of endometriotic lesions shows that abnormal amounts of iron and environmental metals are present [10]. The cause of these deposits has yet to be further investigated, However, they stand out as causal elements in the genesis of endometriotic pathology.

In parallel, from the point of view of genetic factors, other sources report how variants in several genes are present in a significant percentage of patients with endometriosis, many of which involve rare and detrimental alterations [11].

These combined findings confirm the complex interplay of genetic and environmental factors [7]. Such complications relating to the etiopathogenesis of endometriosis make it equally complex to suggest safe preventive methods and to make a positive diagnosis based on definite factors, including those of microbiological or genetic nature. The consequence of this may include a diagnostic delay, which we have already mentioned, an element of frustration for the woman, who may live several years even with a suffering that receives no response and may appear, therefore, insoluble and liable only for non-peaceful cohabitation.

## 3. Chronic Diseases, Endometriosis, and Quality of Life

Health-related quality of life (HRQOL) is defined as “the value assigned to quality of life as modified by impairments, functional states, perceptions and social opportunities influenced by disease, injury, treatment or policy” [12]. This concept includes physical and psychological health, the quality of social relationships and personal independence, beliefs about one’s health, and characteristics of the environment [13].

The impact of the disease on the well-being of affected women is evident, especially if we refer to the World Health Organisation’s definition of ‘health’: “a state of complete physical, mental, psychological, emotional and social well-being” [14].

Women affected by endometriosis report lower scores in various aspects of physical and mental health; physical pain is highlighted as the most relevant in influencing quality of life [15]. However, it is crucial to analyze the individual subjective factors that lead to a different experience of pain. In fact, the intensity of the pain is not directly related to the width and size of the lesions: there are women with profoundly developed endometriosis who report modest symptoms, while it is possible that small lesions can cause very intense pain [4].

Therefore, a complex interaction between quality of life and the psychological aspects related to it clearly emerges: various depressive and anxiety symptoms are reported by patients in the context of impaired quality of life [16,17].

Endometriosis is not only considered one of the costliest public health problems due to the large number of women whom it affects, but its impact on quality of life also relates to the significant morbidity associated with it [18]. In fact, this pathology entails an overall loss of well-being and negatively affects every area of the patient’s life. Let us remember that its main symptoms, such as secondary dysmenorrhea, deep dyspareunia, chronic pelvic pain, and infertility, affect the most fundamental areas of women’s well-being and daily life.

## 4. Quality of Life from a Psycho-Social Perspective

Considering the symptoms reported and observing them from a psychological, emotional, and relational point of view, we can preliminarily assume that it is not uncommon for patients to have difficulty communicating their discomfort. Sometimes, this happens because of a lack of awareness or consciousness of their suffering, due to an underestimation of the problem or fear of not being believed. In fact, it may be particularly burdensome to talk about one’s difficulties in the affective and sexual sphere with one’s family members or with one’s general practitioner because of feelings such as shame or guilt.

With regard to the sexual sphere, in dyspareunia, coitus is frequently associated with persistent and recurrent pain (i.e., it affects genital sensory afferents). On a psychic level, pain may provoke feelings of anger, and the partner may be experienced as the cause of one’s suffering, or the woman may experience intense feelings of sexual and personal inadequacy, with consequent depressive feelings that would then also have an impact on her sexual desire [19]. The theme of difficulties in the sexual sphere, which in itself can constitute a strong element of crisis in the couple, can be considered in conjunction with that of infertility or hypofertility. Dyspareunia and infertility both affect the woman’s self-representation and can lead to depressive feelings, feelings of inadequacy, or guilt. These feelings hinder and make more difficult the necessary work of elaboration, by the woman and the couple, of the mourning linked to the loss of ideals and the redefinition of plans in an adaptive sense.

For these reasons, patients with endometriosis often have a quality of life that is severely compromised by significant physical and psychological suffering due, on the one hand, to persistent symptoms, the side effects of medical therapies, and the outcomes of surgery [18] and, on the other hand, the possible presence of depressive feelings. This status inevitably severely compromises the possibility of a normal working, emotional, and relational life. Chronic illnesses cause significant changes in daily life and in the planning of it. The recurring pain, physical and emotional illness, and the work implications, as well as the economic ones, lead to insecurity in the sufferer and to inquiries not only of the personal but also of the family sphere [13].

Frequently, the diagnosis of endometriosis is reached after a long and costly journey, which is connected with much suffering. During the period of seeking the diagnosis, patients undergo many diagnostic tests and treatments, often unnecessary and without benefit, which in addition to the possibility of causing adverse effects, further increase healthcare costs. In this sense, the inclusion of HRQOL measures in clinical assessment can contribute to an improvement in the diagnostic accuracy and clinical outcomes while promoting public health and feelings of self-efficacy in women with endometriosis [13].

**Figure 1:**
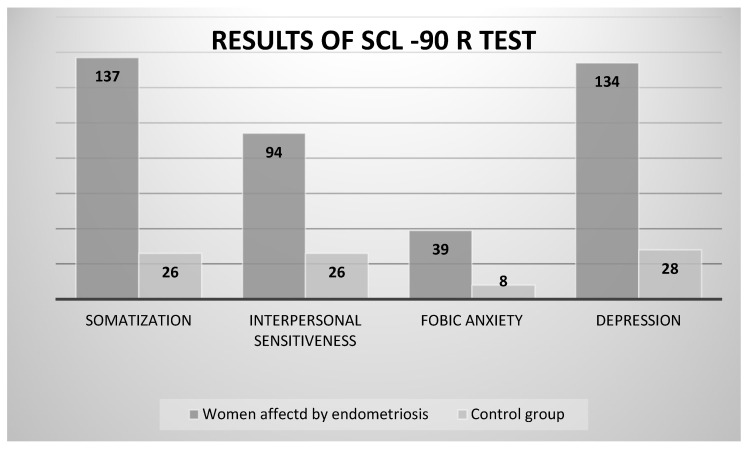
Detailed graph showing the difference between the target group (women with endometriosis) and the control group in some subscales of the SCL-90 R test. Data from Laganà and colleagues [20].

Laganà and colleagues [20] investigated aspects of the quality of life of women with endometriosis and emotions such as anger, anxiety, and depression, in addition to possible psychopathological comorbidities. The group of patients with endometriosis consisted of 166 subjects (mean age: 36 ± 6 years), and there were 48 patients in the control group (mean age: 38.4 ± 12.8 years). The researchers used the SCL-90 R scale (for the self-assessment of psychic symptomatology) and, as can be seen in Figure 1, the scores in the subscales for somatization (*p* = 0.02), depression (*p* = 0.01), interpersonal sensitivity (*p* = 0.04), and phobic anxiety (*p* = 0.04) were significantly higher than in the control group.

**Figure 2:**
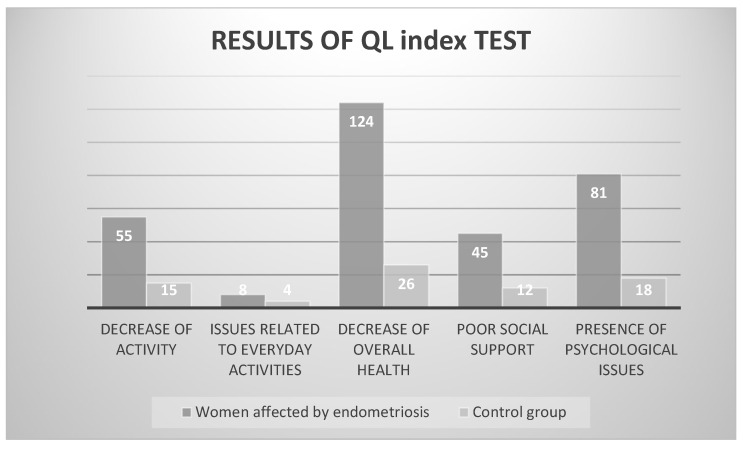
Detailed graph showing the difference between the target group (women with endometriosis) and the control group in the QL Index. Data from Laganà and colleagues [20].

Regarding quality of life (QL index), there was a significant decrease of overall health reported by women with endometriosis compared to the control group (*p* = 0.008), as can be seen depicted in Figure 2. 

## 5. Quality of Life from the Perspective of Sexuality and Relationships

The World Health Organization (WHO) defines sexual health as follows: “Sexual health is a state of physical, emotional, mental and social well-being related to sexuality; not reducible to the absence of disease, dysfunction or infirmity. Sexual health requires a positive and respectful approach to sexuality and sexual relationships, as well as the possibility of having pleasurable and safe sexual experiences, free from coercion, discrimination and violence. For sexual health to be achieved and maintained, everyone’s sexual rights must be respected, protected and fulfilled” [21].

Therefore, it is now well established that sexuality, while being one of the primary and evolutionary impulses that guide human behavior, is a complex phenomenon involving various organic and psychic aspects of the person, as well as aspects that depend on the culture, i.e., the society to which one belongs.

A multidisciplinary approach that takes into account social, educational, hormonal, and biological factors, as well as medical and surgical ones, cannot ignore how endometriosis is a pathology that decisively affects the domains of sexual function and the quality of relationships.

The most common symptoms that characterize endometriosis are chronic pelvic pain, fatigue, congestive dysmenorrhea, heavy menstrual bleeding, and profound dyspareunia. Studies [22,23,24] have shown the significant negative impact of this condition on women’s quality of life (QoL) and their relationships with their partner.

A recent study involving five hundred participants aged 18–63 years shows a significant relationship between endometriosis health profile scores and personal well-being index [25]. The results of the qualitative data suggest that endometriosis has a negative impact on several areas of experience such as social life, romantic and sexual relationships, and future planning. These factors affect women’s overall quality of life by contributing to work productivity problems and social dissatisfaction and by increasing the risk of psychological comorbidities.

Longitudinal studies are still in their infancy and predominantly focused on heterosexual subjects, but despite this, data are already emerging that show how within the relationship, partners are affected by the disease, and how this affects the well-being of the sexual relationship, amongst other factors.

The data emerging from the literature show how, concerning sexual function, women feel that their life is half-full, that they do not function well, and that they are defective. They emphasize, in fact, a decrease in the frequency and quality of complete sexual intercourse after the diagnosis, which, combined with an impairment of overall sexual activity and a drop in self-esteem, leads to an overall decrease in sexual satisfaction.

All of this generates in them a deep sense of inadequacy and guilt and makes them experience the sexual approach of their partner with difficulty. The decline in desire, linked not only to physical pain, but also to the thought and fear of the same, leads them to reject their partner’s approaches and, furthermore, to lose shares of expressed intimacy and affection, for fear of being misinterpreted.

Recent research [26] investigating the impact of endometriosis on the male partner shows that partners have also reported a significant impact of endometriosis on various areas of life, such as on sexuality, intimacy, family planning, as well as on the couple’s financial budget. The risked consequence is the establishment of a situation that sees even the most common and daily affective demonstrations reduced, such as hugging, kissing, and being physically close. On the male side, partners report feeling frustration, occasional anger, and disappointment. These feelings lead in some cases to disturbances even in the male sexual sphere (e.g., decreased libido or erectile dysfunction), as well as relational problems of varying complexity (e.g., an excessive use of computer media for information and communication or infidelity). It is evident how these aspects can only worsen the woman’s quality of life, which is already compromised by a chronic illness, and risk fueling feelings of guilt and shame related to the disease.

As shown by large and recent studies [27,28,29], sexual health is a very important aspect of quality of life and should be considered a goal of endometriosis-related treatment. It is therefore fundamental to take into due consideration the quality of sexual relationships and favor the expression of the woman’s and partner’s needs, involving them in both individual and group therapy.

The reduction in quality of life (QoL) among women with endometriosis is more pronounced in both mental and physical aspects, in contrast to non-endometriosis conditions that seem to impact the physical aspect of QoL to a lesser extent [15]. This condition is connected with decreased mental health in patients, with clear influences on the possibilities for emotional and sexual interactions [15,16].

This extra step could be useful in intervening on emerging feelings in the woman such as frustration, anger, guilt, and shame, as well as allowing for a more open discussion with other women (and partners) on both sexual and relational issues, which are in fact closely linked.

## 6. The Experience of Pain

It appears from the literature that pain is a central component of endometriosis pathology, in particular chronic pelvic pain, a condition that negatively marks the woman’s daily life [20].

Concerning the experience of pain, we will make some observations regarding its quality. In particular, pain originates from tissue damage, which in turn is signaled to the brain by specific pain receptors that signal a difference in state. This physiological process emerges to our conscious attention through an unpleasant sensation, to which a location in the body is attributed, specifically, the area from which the signals came. This allows the individual to act on the source of the pain and possibly eliminate it at the source, since an organic problem could threaten the individual’s survival. While this process may be considered routine, as well as necessary, in an individual’s life course, the psychological experience of pain may be very particular and different for each individual. This is because the experience of pain is shaped by previous experiences [30].

In particular, previous experiences of illness and pain form anticipatory beliefs, i.e., expectations, that shape how people cope with the experiences and, above all, the perceptions themselves [31]. This occurs through memory systems, which store perceptual events, and related emotional reactions. In the early years of an individual’s life, these memories consist of perceptual-motor patterns related to emotional experiences. As the individual grows and acquires language and, therefore, the ability to symbolically represent experiences, he or she will also store related thoughts and images, generating increasingly sophisticated and complex patterns of action and reaction, adapted to the challenges posed by the world. However, the automatic action patterns stored in early childhood at the perceptual-motor (embodied) level remain at a fundamental level of experience.

In other words, the automatic schemata and the beliefs connected to them go on to constitute structures of varying complexity, which, taken together, will constitute the individual’s vision of the world, or rather of oneself in relation to the world, which is therefore never alone, but constitutes all these schemata based on the relationship with the Other, first with the mother (or primary caregiver, who should guarantee its survival) and then in all other significant relationships.

On certain occasions, such as in cases of perceived threats to one’s integrity or in cases of high stress, the basic (or original or primary) schemas of action may come into action, as they are brought on by profoundly intense experiences that call for an immediate and decisive resolution, but, at times, they are not reflective of reality. That is, the solutions adopted (e.g., flight in the form of avoidance) may not accord with the adult’s context, in which the individual lives given organic maturation (e.g., an avoidance of a clinical examination for fear of the outcome).

This leads to intense emotional experiences and feelings (including painful ones) due to the intolerability of the situation for the individual, subjectively perceived and evaluated. In other words, the psychological experiences do not accord with the real threats that are present to the individual, be they external situations or those that are inherent to organic suffering. These elements, deduced from the literature, outline how the functioning of neural networks defines the psychological experience of the individual through, in particular, attentional processes. Attention, in fact, constitutes the instrument for directing energies and actions aimed at resolving the current organic problem that, to the mind, emerges as pain. Pain constitutes an imbalance in psycho-somatic homeostasis, to which a response must be given (for the adaptive reasons mentioned above). If, however, previous experiences have already directed the way in which an individual experiences and reacts to pain, the attentional allocation may be either misdirected or maintained for an excessive amount of time, or the attentional load may be excessive in relation to the actual pain stimulus [32].

The result of such perturbations of attentional aspects could result in an altered experience of pain, i.e., in unpleasant sensations that are disproportionate to the actual injury, or the pain sensations could require attentional resources (which would generate stress, in physiological and psychological terms) to be maintained for an excessive amount of time, or the attentional resources could be concentrated on body districts that are inconsistent with the injuries.

A distinction must therefore be made between somatic physiology, perceptual experience, the related interpretative schemes (of increasing complexity as development proceeds), and, finally, schemes of reaction to stimuli. While the first components of this procedural chain are organic and physiological (inherent to shared human anatomy), the interpretative and reaction patterns are certainly individual [30].

## 7. Pain and Quality of Life: The Psychological Impact in Endometriosis

The point that is important to emphasize for our purposes is that in all chronic pathologies, not only does the experience of pain correlate negatively with the reported quality of life, but it also appears that the organic aspects of the disease correlate extremely poorly with the aspects relating to disability, quality of life, and stress [32], highlighting the importance of paying particular attention to the psychological and social factors of the disease [33]. The subjective element of the disease, based, as mentioned, on the ontogenetic development of the individual (and on cultural aspects), would therefore be ubiquitous and at the same time particular to each individual and would mark the experience of the disease and specifically the quality of life in a peculiar way (as we observed in the case of women with endometriosis in Laganà and colleagues [20]).

The importance of the assessment of non-organic symptoms of a psychological nature also emerges from the suffering brought forward by patients who come for medical assessment, which cannot, however, be biologically explained in its entirety by physiological elements, with at least 33% of somatic symptoms being classified as medically unexplained [34]. Several sources in the literature [30,35,36,37,38,39,40], consistently, show how (in the case of endometriosis, but also other chronic disorders) psychological interventions in conjunction with medical interventions that are tailored to the specific problem and the severity of the problem, lead to a reduction in psychological suffering (related to stress, anxiety, and depression), as well as a reduction in perceived pain.

In the specific pain experience associated with endometriotic lesions, two aspects are typically present: hyperaesthesia, i.e., increased sensitivity in one body district, and hyperalgesia, i.e., an experience of intense pain in one body district [41,42]. These aspects are included in the paresthetic conditions, which are characterized by an altered perception of sensitivity to sensory stimuli, including pain stimuli. This means that a proportion of the pain experienced by women with endometriosis (which varies depending on the individual) may be attributable to an erroneous attentional attribution in the face of a real and concrete pain experience which, precisely because it is chronic, leads to physiological processes of proliferation of the pain nerve endings. At the same time, however, an investment of important attentional resources takes place, subtracting them from other processes and further amplifying the presence of unpleasant sensations in the field of conscious awareness.

With this brief exposition on pain and the existing literature, we therefore wish to acknowledge the complexity of the experience of women with endometriosis, whose bodily pain is correlated with very complex and multifaceted psychological experiences that are inherent to living a daily life that is full of suffering and limitations. Furthermore, the essential elements related to sexuality, marital life, and family planning are significantly (and at times lastingly) impacted due to both the lesions and the encounter with pain. In this sense, organic pain appears as a single component of a more complex somato-psychic condition, as is also evident from the results of the SCL-90 administered by Laganà and colleagues [20], where very high scores were attributed to the symptomatological dimensions of somatization, depression, and sensitivity, or to the STAXI-2 scale administered as part of the same study, where scores relating to the state of anger and the expression of anger emerged [20].

The psychological experience of pain, therefore, even if for most people it is different, complex, and most commonly episodic, in women with endometriosis, it becomes a daily topic and demands considerable amounts of psychic energy. Addressing, within the framework of a psychological pathway, this experience could not only propose different ways of coping, but above all revise beliefs and expectations relating to pain, allowing the woman to modify automatic patterns of reaction that lead to the reiteration of strategies that are a source of frustration.

## 8. Conclusions

We highlighted the complexity of endometriosis’s pathology, the etiopathological mechanisms of which are still being studied and discovered. However, a picture is emerging, in which this pathology appears to originate and be maintained by a complex of integrated genetic, environmental, and psychological factors.

While from the point of view of genetic and environmental factors, several attempts are being made to help prevent and intervene in endometriosis, from the psychological point of view, the factors that worsen women’s QoL are highlighted but not yet circumscribed, even given the complexity of the pain experience.

We therefore stress how relevant it may be to investigate the experience of women with endometriosis from a psychological point of view in light of the latest neuropsychological research, enabling us to specify modes of thinking and pain management, being able, consequently, to calibrate specific psychological interventions. In fact, we consider the chronicity that characterizes this pathology to be a factor of particular distress, one that needs to be tended to, especially until alternative and more effective medical therapies than the current ones emerge.

Research may aim, in the future, to investigate the complex relationships between the experience of the disease, the experience of pain, and women’s psychoemotional expression, thus contributing to the treatment of one of the various factors that constitute, in an integrated and complex way, the evolution and course of endometriosis’s pathology.

## Data Availability

No new data were created or analyzed in this study. Data sharing is not applicable to this article.
